# Aurantiogliocladin inhibits biofilm formation at subtoxic concentrations

**DOI:** 10.3934/microbiol.2017.1.50

**Published:** 2017-01-24

**Authors:** Kamila Tomoko Yuyama, Thaís Souto Paula da Costa Neves, Marina Torquato Memória, Iago Toledo Tartuci, Wolf-Rainer Abraham

**Affiliations:** Helmholtz Center for Infection Research, Chemical Microbiology, Inhoffenstrasse 7, 38124 Braunschweig, Germany

**Keywords:** biofilm, fungi, *Clonostachys candelabrum*, biofilm inhibition, minimal inhibitory concentration, *Staphylococcus epidermidis*, quorum-quenching activity

## Abstract

Infections where pathogens are organized in biofilms are difficult to treat due to increased antibiotic resistances in biofilms. To overcome this limitation new approaches are needed to control biofilms. One way is to screen natural products from organisms living in a wet environment. The rational is that these organisms are preferentially threatened by biofilm formation and may have developed strategies to control pathogens in these biofilms. In a screen of fungal isolates obtained from the Harz mountains in Germany several strains have been found producing compounds for the inhibition of biofilms. One of these strains has been identified as *Clonostachys candelabrum* producing aurantiogliocladin. Biological tests showed aurantiogliocladin as a weak antibiotic which was active against *Staphylococcus epidermidis* but not *S. aureus*. Aurantiogliocladin could also inhibit biofilm formation of several of the tested bacterial strains. This inhibition, however, was never complete but biofilm inhibition activity was also found at concentrations below the minimal inhibitory concentrations, e. g. *Bacillus cereus* with a MIC of 128 µg mL^−1^ showed at 32 µg mL^−1^ still 37% biofilm inhibition. In agreement with this finding was the observation that aurantiogliocladin was bacteriostatic for the tested bacteria but not bactericidal. Because several closely related toluquinones with different antibiotic activities have been reported from various fungi screening of a chemical library of toluquinones is suggested for the improvement of biofilm inhibition activities.

## 1. Introduction

In many infections, pathogens are embedded in a complex matrix of macromolecules that adhere to various surfaces, a lifestyle called biofilm. Biofilm infections cause severe complications and 12% to 25% of bloodstream infections are attributable to patient mortality [1]. Biofilms form on any implant but also on mucosa, e. g. in the cystic fibrosis lung [2], the middle ear [3], the urinary tract [4] or gastric mucosa [5]. Biofilm formation protects the embedded microbial cells against hostile environments, including antibiotics or the immune system of the host. Biofilms are dynamic, adapting to changes in the environment and biofilm formation is reversible, enabling cells to disperse and to colonize new habitats if the conditions become unfavorable [6].

To form a biofilm, microbes have to coordinate their activities. This communication is achieved by small molecules acting as autoinducers to start genetic programs [7]. If the autoinducer concentration reaches a certain threshold, biofilm formation starts followed by the production of virulence factors. Because the process depends on cell density, this is called quorum sensing and used by many microbes [8]. To date more than 100 different autoinducers are known for bacteria, archaea and fungi. The best studied autoinducers are N-acyl-L-homoserine lactones, only known from Gram-negative bacteria [9]. Autoinducer-2, having a rather unusual cyclic boronic ester, is known both from Gram-positive and -negative bacteria [10]. In many Gram-positive bacteria small peptides act as autoinducers which are derived from larger precursor peptides [11].

The eradication of biofilm infections is a difficult task because the embedded pathogens are well protected against macrophages and antibiotics [12], e.g. 220 times higher antibiotic concentrations were needed to kill *Escherichia coli* in a biofilm compared to cells of the same strain living in planktonic form in the serum [13]. To overcome these difficulties together with increasing antibiotic resistances [14] novel antibiotics are needed to control biofilm infections [15]. One of these approaches aims at the communication between the cells in a biofilm by blocking quorum sensing [16],[17]. On the one hand compounds from large chemical libraries can be screened but on the other hand natural compounds from various organisms can be tested for their ability to prevent biofilm formation or to disperse existing ones. The rationale behind this approach is that all organisms have to deal with the formation of biofilms on their bodies and probably have developed strategies to fight pathogenic biofilms. One potential resource for novel biofilm-modulating compounds are sporocarps of fungi which are exposed to a wet climate, favorable conditions for biofilm formation. As a proof of this concept it has been demonstrated for sporocarps of Basidiomycotina that ectomycorhizal fungi had a higher diversity of bacterial biofilms on their fruiting bodies than saprophytic ones which showed a higher percentage of biofilm-inhibiting extracts than ectomycorrhizal fungi [18].

## 2. Materials and Method

### 2.1. Chemicals and reagents

Acetonitrile, ethyl acetate, chloroform and methanol were purchased from J. T. Baker (USA, Germany and Netherlands respectively), d-chloroform, formic acid 98%, potato dextrose (PD) and Luria-Bertani broth (LB) from Carl Roth GmbH (Karlsruhe, Germany). Bacto malt extract, Bacto peptone and agar were from BD (La Point de Claix, France), D-glucose from Merck (Darmstadt, Germany). N-hexanoyl-L-homoserine lactone was purchased from Cayman Chemical Company (USA), crystal violet from Fluka (Steinheim, Germany), tetracycline and RPMI 1640 medium from Sigma Aldrich (Germany, United Kingdom), respectively.

### 2.2. Microorganisms

*Bacillus cereus* DSM 626, *Staphylococcus epidermidis* ATCC 35984, *Streptococcus mutans* UA59, *Staphylococcus* aureus DSM 1104, *Candida albicans* DSM 11225, *Candida guilliermondii* DSM 70052, *Candida krusei* DSM 6128, *Candida parapsilosis* (DSM 5784, *Rhodotorula glutinis* DSM 70398 and *Yarrowia lipolytica* DSM 70151 were purchased from the German collection of microorganisms and cell cultures (DSMZ). Fungi were used in antifungal assays. The bacteria and the mutant *Chromobacterium violaceum* CV026; *Pseudomonas aeruginosa* PA14 and *Escherichia coli* MT102 were applied in antimicrobial and antibiofilm assays. Bacterial strains were maintained on LB agar, and yeast strains on YM agar (0.3% yeast extract, 0.3% malt extract, 0.5% peptone, 1% glucose, 1.5% agar) at 4 °C.

### 2.3. Isolation and strain identification

Fungi were collected in Harz mountains (latitude 51°45′21″, longitude 10°32′17″, 724 m), Germany, and isolated on Potato Dextrose Agar (PDA). The identification of the isolates were made by sequencing of the internal spacer regions (ITS). For DNA extraction, the NucleoSpin Plant II kit (Macherey, Nagel) was used. Fungal primers ITS1F and ITS4 were applied in PCR amplification following Bruns et al. [19]; the amplicons were sequenced by Eurofins, analyzed using Sequencher 4.10.1 and the phylogenetic tree was constructed with the MEGA 6 program. The sequence (LT635436) was deposited in European Nucleotide Archive (ENA).

### 2.4. Fermentation

Pellets (5 × 5 mm) of mycelia of the isolates grown on Malt extract agar (3% malt extract, 0.5% bacto peptone and 1.5% agar) were transferred to 2L Erlenmeyer flasks containing 1L of malt extract (ME) broth (3% malt extract, 0.5% bacto peptone). After 65 days of static incubation at 20 °C or 25 °C in the dark, the broth was filtrated, extracted with ethyl acetate and tested for antimicrobial and antibiofilm activities.

### 2.5. Purification and structure elucidation

Aurantiogliocladin was purified in a gradient with an Agilent HPLC (C18 column Varian, Agilent Technologies 250 × 10 mm). Solvent A: acetonitrile, solvent B: MilliQ® water with 1% formic acid, gradient from 10% A at 0 min to 100% A at 25 min; UV absorbance was detected at 210, 230, 254, 260 and 280 nm; flow rate: 4 mL min^−1^. Molecular mass was identified in Agilent LC-MS/MS 1200 series system (C18 analytical column Nucleosil® 125 mm × 20 mm), with the same conditions described above. The structure was elucidated by ^1^H and ^13^C NMR recorded on a Bruker 600 MHz spectrometer, in 5 mm tube of d-chloroform. The chemical shifts were compared with the literature.

### 2.6. Determination of quorum quenching activity

After overnight incubation at 30 °C in LB medium, *C. violaceum* CV026 was adjusted to OD 0.5 according to McFarland scale, mixed with 3.5 nmol mL^−1^ *N*-hexanoyl-L-homoserinelactone, and inoculated in 96 well no tissue microtiter plates (Falcon®Micro TestTM, USA) containing LB broth with serial diluted aurantiogliocladin (180 to 3 µg mL^−1^) dissolved in methanol. Plates were covered with a sterile adhesive porous paper (Kisker Biotech GmbH, Steinfurt, Germany) and incubated at 30 °C for 48 h. Methanol and LB broth were used as negative and tetracycline (100 µg mL^−1^) was used as positive control. Absence of violacein indicated quorum-quenching activity.

### 2.7. Antimicrobial, antifungal and biofilm inhibition activities of aurantiogliocladin

To determine the minimal inhibitory concentration (MIC) of the bioactive compound, bacteria and yeast were incubated in LB and in RPMI 1640 media respectively, *C. violaceum*, *B. cereus*, *S. epidermidis* and all yeasts at 30 °C and the other strains at 37 °C, containing serial diluted aurantiogliocladin (256 to 3 µg mL^−1^) dissolved in methanol. Bioscreen-C automated growth curve analysis system (Oy Growth Curves AB Ltd, Helsinki, Finland) was used to determine the bacterial/fungal growth over 24 h, employing one OD600 measurement each 15 min. Experiments were performed in triplicate. To evaluate bactericide/fungicide or bacteriostatic/fungistatic effects, aliquots from different concentrations in the well, after OD measurements, were inoculated in LB agar for bacterial assays and YM agar for fungal assays and incubated for 24 h. Methanol and LB/RMPI 1640 media were used as negative, tetracycline (100 µg mL^−1^) as positive control for bacteria.

For biofim inhibition bacteria were incubated as above. After 24h, biofilm formation in the microtiter wells was measured by the staining with crystal violet following a published protocol (O'Toole, 2011) [20].

## 3. Results

**Figure 1.**
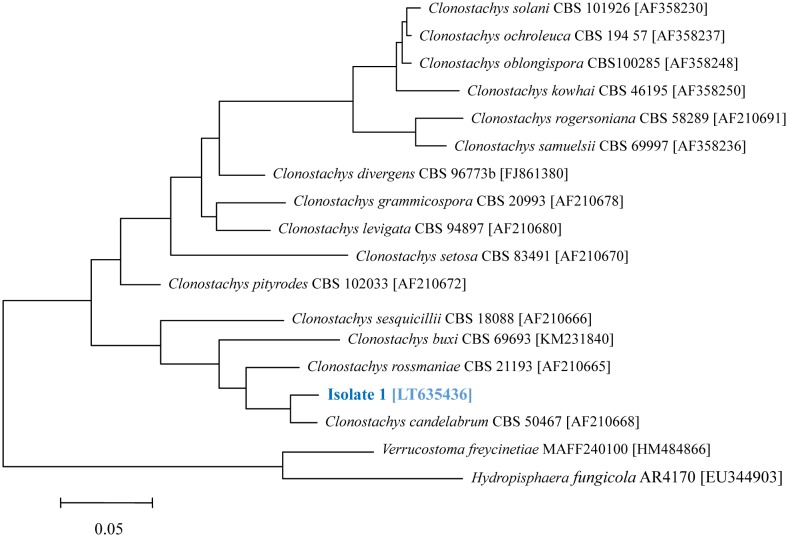
Maximum-likelihood tree constructed with ITS sequences, showing the phylogenetic position of isolate 1 [LT635436] within the genus *Clonostachys*. The sequences of *Verrucostoma freycinetiae* and *Hydropisphaera fungicola* was used as outgroups. Acc. no. are given in square brackets. Bar, 0.05 substitutions per nucleotide position.

To assess the potential of fungi collected at the Harz Mountains, Germany, several isolates were screened for their potential to infer with bacterial biofilm formation. Among the fungi, showing activities, isolate 1 was selected for further investigation of compounds against biofilms of Gram positive and negative bacteria. The isolate was identified by comparison of its ITS region with that of related type species and the sequence was deposited (LT635436). According to the sequence of the ITS region, isolate 1 belongs to the genus *Clonostachys* and is closely related to *Clonostachys candelabrum* (Figure 1).

**Figure 2.**
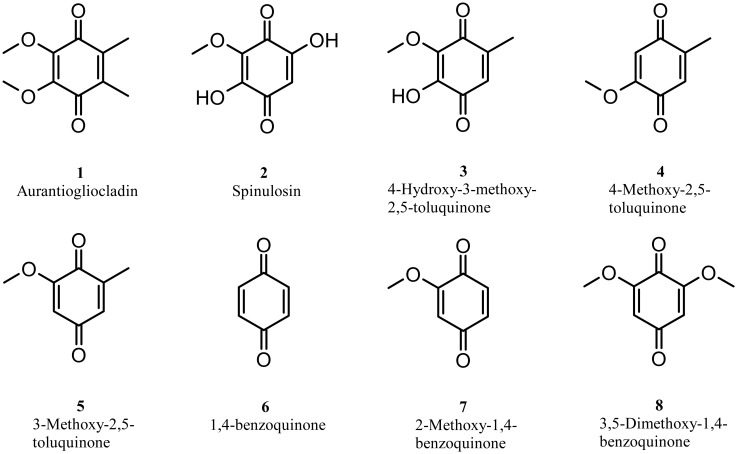
Structures of aurantiogliocladin and related compounds.

From the culture broth of isolate 1 [LT635436] a compound was isolated which formed yellow crystals. It revealed in the ESI mass spectrum a [M + H]^+^ ion of 197.0822 in the positive mode which led to a molecular formula of C_10_H_12_O_4_. Such a molecular composition requires five double bond equivalents. The ^1^H NMR spectra had only two singlets at 2.05 and 3.98 (CDCL_3_) while the ^13^C NMR spectra displayed the resonances of two carbonyls and two double bonds demanding one ring to fulfill the molecular formula. The spectral data and comparison with literature data [21] led to the identification of the active compound as aurantiogliocladin 1 (Figure 2). Aurantiogliocladin has been discovered in the culture broth of a *Gliocladium* species [22] and its structure was determined in 1953 by Vischer [23]. Biological tests showed that it is a weak antibiotic but no activities against biofilms have been reported.

**Figure 3.**
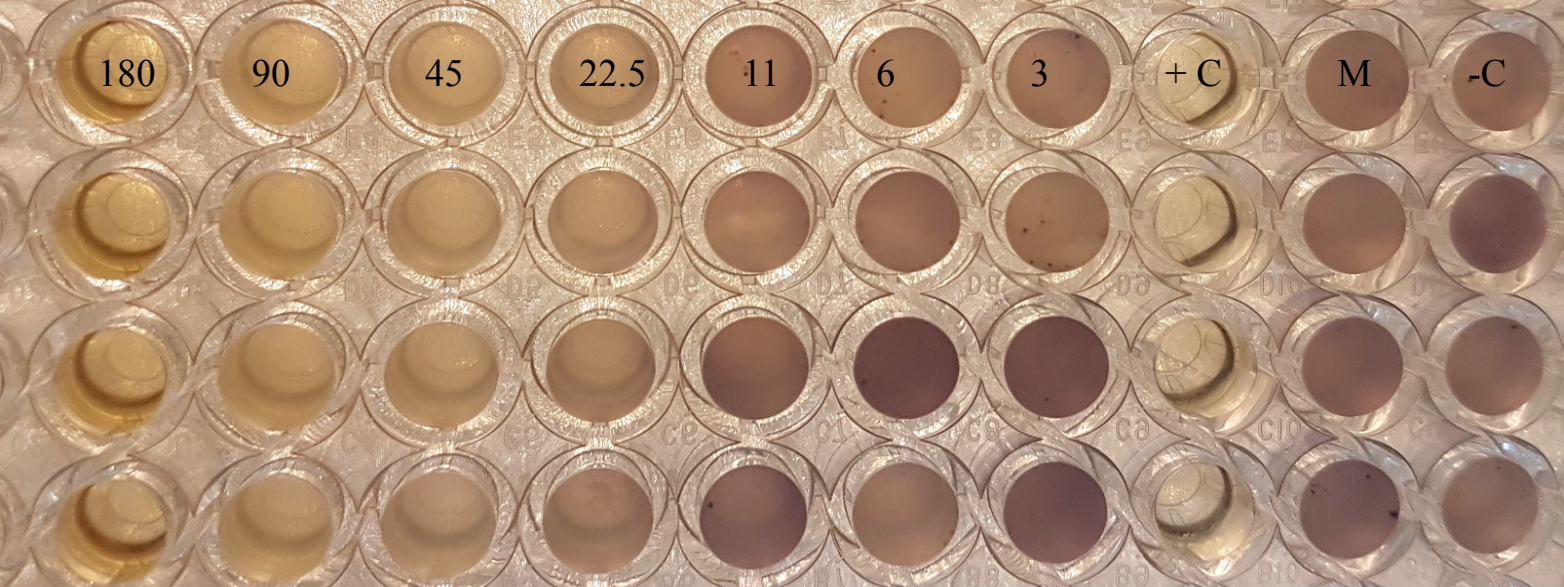
Quorum-quenching activity from aurantiogliocladin against *C. violaceum* CV026. Each column has four replicates of aurantiogliocladin concentrations (180, 90, 45, 22.5, 11, 6 and 3 µg mL^−1^) and controls. +C: Positive control with tetracycline (100 µg mL^−1^). M: Negative control with methanol 3%. –C: Negative control with CASO medium.

The antibiotic activity of aurantiogliocladin against several bacteria was low to moderate. No clear tendency could be seen but Gram-negative bacteria seemed to be more resistant against aurantiogliocladin than Gram-positive. *S. epidermidis* was found to be more sensitive against the antibiotic than the closely related *S. aureus* (Table 1). These differences were more pronounced in the biofilm inhibition assay. While for *S. epidermidis* only 51% biofilm inhibition was observed at MIC concentration (64 µg mL^−1^) and half of the MIC prevented only 19% biofilm formation, *B. cereus* revealed 44% biofilm inhibition at MIC but still 37% at one quarter of MIC. Both, *S. epidermidis* and *B. cereus*, still formed almost 50% of the biofilm when twice or even higher concentrations of MIC were applied. The most sensitive strain in the test was *C. violaceum* CV026 where 90% of the biofilm could be prevented at MIC and still 29 % at one eighth of MIC. These results indicate that biofilm inhibitions were almost independent from the minimal inhibition concentrations for planktonic cells, and also were associated with quorum-quenching activity (180–22.5 µg mL^−1^) due to the absence of violacein (Figure 3). Interesting is the finding that some inhibition of biofilm formation occurred even at subtoxic concentrations of aurantiogliocladin. To shed more light on these results bacteria we treated with aurantiogliocladin at MIC and were then plated on fresh agar plates. All bacteria grew again under these conditions indicating that aurantiogliocladin is not bactericidal at this concentration but bacteriostatic.

**Table 1.**
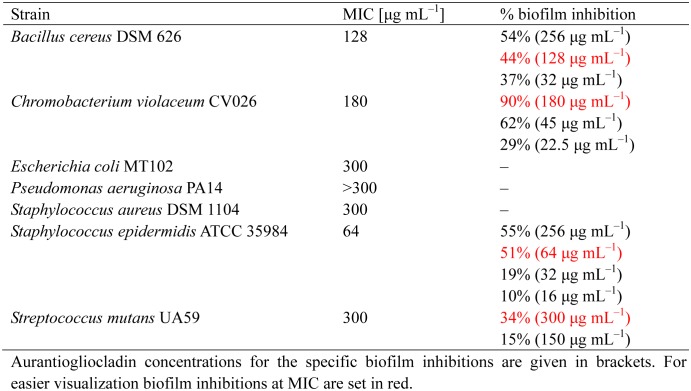
Antibacterial activities of aurantiogliocladin.

Because in the first publications of aurantiogliocladin activities against fungi have been reported we also tested the antifungal activity of this compounds against several yeasts (Table 2). Aurantiogliocladin was moderately active against all yeasts tested with the highest activities against *C. guilliermondii* and *R. glutinis* and the lowest activity against *C. albicans*. As already found for the tested bacteria at MIC the substance was fungistatic but not fungicidal.

**Table 2.** Antifungal activities of aurantiogliocladin.

Strain	MIC [µg mL^−1^]
*Candida albicans* DSM 11225	128
*Candida guilliermondii DSM 70052*	32
*Candida krusei* DSM 6128	64
*Candida parapsilosis* DSM 5784	64
*Rhodotorula glutinis* DSM 70398	32
*Yarrowia lipolytica* DSM 70151	64

## 4. Discussion

Aurantiogliocladin 1 is known for several decades and has been found in a number of strains. Various studies were directed towards the biosynthesis of aurantiogliocladin and it was found that it starts from acetate and runs via orsellic acid to the final metabolite [24]. 1,3-dihydroxy-4,5-dimethylbenzene, also reported from *Gliocladium roseum* maybe a precursor for the intermediate orsellic acid [25]. Aurantiogliocladin was discovered as a weak antibiotic but later found to be also weakly anthelmintic [26] and toxic for termites (*Coptotermes formosanus*) [27] and also for the second instar larvae of *Aedes aegypti* mosquito [28]. Furthermore it serves as attractant for the plant-parasitic nematode *Neotylenchus linfordi* which is associated with *Gliocladium roseum* [29].

Secondary metabolites with a quinone skeleton were described from several fungi. One of the first reports of a compound of this class was spinulosin 2 from *Penicillium spinulosum* [30]; from *Aspergillus fumigatus* several toluquinones have been reported, e. g. 4-hydroxy-3-methoxy-2,5-toluquinone 3, which are closely related to aurantiogliocladin [31]. 4-Methoxy-2,5-toluquinone 4, isolated from *Lentinus adhaerens*, has been shown to block the thromboxane A_2_ receptor [32]. Not only in fungi but also in animals this type of secondary metabolites can be found. The millipede *Floridobolus penneri* excretes for defense a number of benzoquinone which are closely related to aurantiogliocladin [33].

Aurantiogliocladin is a weak antibiotic acting both against a number of bacteria but also against some fungi. The selectivity of the antibiotic is remarkable. While *S. aureus* was not inhibited at all, *S. epidermidis* was more sensitive. The compound had not influence on the growth of *P. aeruginosa* or *E. coli*. In all these experiments it was found that plating on fresh medium after the inhibition with aurantiogliocladin resulted in the growth of the treated cells, demonstrating that the antibiotic stalls growth but does not kill the cells. This phenomenon has already been reported for less substituted 1,4-benzoquinone and *S. aureus* [34]. The action on biofilm formation is more complex. For some bacteria there are only little effects on the biofilm (e. g. *S mutans* or *S. epidermidis*), while for other strains biofilm inhibition is considerable large (e. g. *C. violaceum*) and lasting well below the MIC threshold (e. g. *B. cereus*).

Some insights into the structure-activity-relations of this class of compounds are gained by comparing literature data of related compounds. Both 4-methoxy-2,5-toluquinone 4 [35] and 3-methoxy-2,5-toluquinone 5 [36] are more active, especially against *S. aureus* and *B. subtilis* than aurantiogliocladin. A study assessing a large number of 1,4-benzoquinones and 2,5-toluquinones demonstrated a low sensitivity of *E. coli* against almost all compounds while the sensitivity of *S. aureus* varied across the different compounds. As tendencies, it was noted that the introduction of methoxy groups at the nucleus increased activity while hydroxyl had the opposite effect. The most active compounds were found to have a methoxy group at C-4 of 2,5-toluquinone and the author recommended 4,6-dimethoxy-2,5-toluquinone derivatives as the most active ones [37]. From these results one can speculate that the additional methyl group and the adjacent methoxy functions cause the decline in activity against *S. aureus*. Interestingly, another study could not find any activities (>800 mg mL^−1^) of 1,4-benzoquinone 6, 2-methoxy-1,4-benzoquinone 7 and 3,5-dimethoxy-1,4-benzoquinone 8, confirming the very different action of this class of compounds on fungi compared to bacteria [38]. None of these studies included biofilms and as the results of the presented study shows MIC data are here a very poor indicator for the prediction of biofilm inhibition.

## 5. Conclusion

The rational that natural products from organisms living in a wet environment may be active against biofilms could be confirmed and fungi proved to be a rich source for biofilm-interfering compounds. In a screen of fungal isolates obtained from the Harz mountains in Germany several strains have been found producing compounds for the inhibition of biofilms. One of these strains has been identified as *C. candelabrum* producing aurantiogliocladin. Biological tests showed aurantiogliocladin as a weak antibiotic which was active against *S. epidermidis* but not *S. aureus*. Aurantiogliocladin could also inhibit biofilm formation of several tested bacterial strains. This inhibition, however, was never complete and biofilm inhibition activities were also found at concentrations below the minimal inhibitory concentrations. In agreement with this finding was the observation that aurantiogliocladin was bacteriostatic for the tested bacteria but not bactericidal. Many quorum-quenching compounds do not kill bacteria and it has been suggested to combine them with antibiotics for improved clearance of biofilm infections [39]. Because several closely related toluquinones with different antibiotic activities have been reported from various fungi, screening of a chemical library of toluquinones is suggested for the improvement of biofilm inhibition activities. These investigations should not be conducted only on pure strains because in nature microbes seldom live alone but in polymicrobial communities with complex and multifold interactions [40]. Any applicable drug for the control of biofilm infections has to address this issue in order not to suppress one pathogen but foster the development of another one in the same biofilm community.
